# Immunophenotypes of anti-SARS-CoV-2 responses associated with fatal COVID-19

**DOI:** 10.1183/23120541.00216-2022

**Published:** 2022-12-05

**Authors:** Julij Šelb, Barbara Bitežnik, Urška Bidovec Stojković, Boštjan Rituper, Katarina Osolnik, Peter Kopač, Petra Svetina, Kristina Cerk Porenta, Franc Šifrer, Petra Lorber, Darinka Trinkaus Leiler, Tomaž Hafner, Tina Jerič, Robert Marčun, Nika Lalek, Nina Frelih, Mojca Bizjak, Rok Lombar, Vesna Nikolić, Katja Adamič, Katja Mohorčič, Sanja Grm Zupan, Irena Šarc, Jerneja Debeljak, Ana Koren, Ajda Demšar Luzar, Matija Rijavec, Izidor Kern, Matjaž Fležar, Aleš Rozman, Peter Korošec

**Affiliations:** 1 University Clinic of Respiratory and Allergic Diseases, Golnik, Slovenia; 2 Medical Faculty, University of Ljubljana, Ljubljana, Slovenia; 3 Biotechnical Faculty, University of Ljubljana, Ljubljana, Slovenia; 4 Faculty of Pharmacy, University of Ljubljana, Ljubljana, Slovenia; 5 These authors contributed equally

## Abstract

**Background:**

The relationship between anti-SARS-CoV-2 humoral immune response, pathogenic inflammation, lymphocytes and fatal COVID-19 is poorly understood.

**Methods:**

A longitudinal prospective cohort of hospitalised patients with COVID-19 (n=254) was followed up to 35 days after admission (median, 8 days). We measured early anti-SARS-CoV-2 S1 antibody IgG levels and dynamic (698 samples) of quantitative circulating T-, B- and natural killer lymphocyte subsets and serum interleukin-6 (IL-6) response. We used machine learning to identify patterns of the immune response and related these patterns to the primary outcome of 28-day mortality in analyses adjusted for clinical severity factors.

**Results:**

Overall, 45 (18%) patients died within 28 days after hospitalisation. We identified six clusters representing discrete anti-SARS-CoV-2 immunophenotypes. Clusters differed considerably in COVID-19 survival. Two clusters, the anti-S1-IgG^lowest^T^lowest^B^lowest^NK^mod^IL-6^mod,^ and the anti-S1-IgG^high^T^low^B^mod^NK^mod^IL-6^highest^ had a high risk of fatal COVID-19 (HR 3.36–21.69; 95% CI 1.51–163.61 and HR 8.39–10.79; 95% CI 1.20–82.67; p≤0.03, respectively). The anti-S1-IgG^highest^T^lowest^B^mod^NK^mod^IL-6^mod^ and anti-S1-IgG^low^T^highest^B^highest^NK^highest^IL-6^low^ cluster were associated with moderate risk of mortality. In contrast, two clusters the anti-S1-IgG^high^T^high^B^mod^NK^mod^IL-6^low^ and anti-S1-IgG^highest^T^highest^B^high^NK^high^IL-6^lowest^ clusters were characterised by a very low risk of mortality.

**Conclusions:**

By employing unsupervised machine learning we identified multiple anti-SARS-CoV-2 immune response clusters and observed major differences in COVID-19 mortality between these clusters. Two discrete immune pathways may lead to fatal COVID-19. One is driven by impaired or delayed antiviral humoral immunity, independently of hyper-inflammation, and the other may arise through excessive IL-6-mediated host inflammation response, independently of the protective humoral response. Those observations could be explored further for application in clinical practice.

## Abstract 2

**By employing unsupervised machine learning, this study identified multiple anti-SARS-CoV-2 immune response profiles and observed major differences in #COVID19 mortality between these profiles** https://bit.ly/3SgMh6n

## Introduction

SARS-CoV-2 can cause a range of clinical manifestations, from asymptomatic to severe acute respiratory disease, and several studies have suggested that dysregulation of innate and adaptive immune responses is likely to contribute to disease severity [1–9]. Induction of more potent neutralising antibodies during SARS-CoV-2 infection has been shown to predict COVID-19 survival [2], and delayed antibody response correlated with severe disease [9]. T-cells are key orchestrators of antiviral immune responses [10], and an imbalance of regulatory and cytotoxic SARS-CoV-2-reactive CD4^+^ T-cells have been demonstrated in hospitalised COVID-19 patients [4]. Recruitment and expansion of T-cells in the alveolar space of COVID-19 patients [11] and other affected organs, such as in the cerebrospinal fluid of neuro-COVID-19 patients [12], further suggests the importance of T-cell response. Consequently, it has been observed that COVID-19 is accompanied by lymphocytopenia [13] and decreases in circulating T-cells [3, 5, 14–17]. Uncontrolled inflammation may also contribute to COVID-19 severity [7]. This hypothesis is consistent with increased C-reactive protein, ferritin, D-dimer, cytokines and chemokines [1, 3, 5, 8, 13]. Notably, high serum interleukin-6 (IL-6) and tumour necrosis factor-α (TNF-α) levels remained independent and significant predictors of patient survival, also when adjusted for clinical severity factors [1]. However, the relationship between humoral responses, T-cell responses and uncontrolled inflammation among patients with COVID-19, and how patterns of these responses affect the trajectories of COVID-19 are poorly understood.

To bridge this gap, we aimed to use unsupervised machine learning to identify the immunological properties of fatal COVID-19 and thus get a better understanding of the fatal disease. Since in unsupervised machine learning (compared to supervised learning) the outcome of interest is hidden to the algorithm at the time of algorithm training, there is no risk to over fit the model to the outcome of interest. Therefore the detected patterns are likely the result of some fundamental biological property of the disease thus giving a unique insight into pathophysiology and possible treatment venues of the disease.

We hypothesised that there are multiple (immune)phenotypes of humoral [2, 9], lymphocyte [3–5, 11–17] and inflammatory [1, 3, 5, 8, 13] immune responses to SARS-CoV-2 that differ in their relationship to outcome trajectories of COVID-19 and that unsupervised data-driven techniques may help identify such immunophenotype patterns by determinants of IgG antibodies to SARS-CoV-2 anti-spike 1, quantitative lymphocyte subsets and IL-6 levels. To address our hypothesis we characterised those immune patterns in a longitudinal hospitalised prospective cohort of patients with COVID-19 and then investigated the association of these patterns with mortality within 28 days after hospitalisation. We also included 40 non-hospitalised subjects with prior COVID-19 infection to serve as (recovered) controls.

## Methods

### Study design and participants

#### Hospitalised study subjects

A prospective observational study of COVID-19 was carried out during the second pandemic wave (September–December 2020) of SARS-CoV-2 in Slovenia. We included consecutively admitted adults with nasopharyngeal swabs who tested positive for SARS-CoV-2 by reverse transcriptase quantitative polymerase chain reaction (PCR). All patients or their legally authorised representatives provided informed consent. Of 268 patients, 14 were excluded because they had already died or had been discharged after inpatient admission within 48 h after the presentation. In addition to patient's requirements for supplemental oxygen at admission and/or radiological signs of COVID-19 pneumonia, the following criteria were adhered to when deciding whether to admit the patient to the hospital: 1) age ≥65 years; 2) body mass index (BMI) ≥30; 3) presence of chronic diseases including chronic kidney disease, diabetes, cardiovascular diseases, chronic lung diseases (asthma, COPD, interstitial lung diseases, *etc*.), cancer, advanced liver disease, *etc*.; and 4) presence of immune insufficiency (*i.e.* recent chemotherapy, patients on certain immunosuppressants, patients with transplantation, *etc*.).

Thus we longitudinally profiled the immune responses against SARS-CoV-2 in 254 hospitalised COVID-19 patients. The total number of collected blood samples was 698, with a median of 2 (IQR 4) samples per patient in an interval of a median of 3 (IQR 4) days (table 1, supplementary figures E2A–G). The median time of hospitalisation was 8 days (IQR 8), and none of the patients was transferred to another facility (table 1). Data on demographics, including age and sex, comorbidities, clinical signs, interventions and outcomes are described in table 1 and the online supplementary material.

**TABLE 1:** Characteristics of 254 hospitalised COVID-19 patients according to mortality at 28 days or to cluster assignment

**Characteristic**	**Died at 28** **days**	**Recovered^#^**	**Cluster 1**	**Cluster 2**	**Cluster 3**	**Cluster 4**	**Cluster 5**	**Cluster 6**
**Subjects n or n (%)**	45	209	51 (20.1)	21 (8.3)	35 (13.8)	15 (5.9)	82 (32.3)	50 (19.7)
**Age**								
Mean±sd years^****; ++^	81.6±8.1	69.7±14	75.3±14.0	77.0±11.1	74.2±11.1	71.8±15.4	70.0±15.6	67.5±11.8
Distribution n (%)^****; +^								
<70 years	5 (11)	93 (44)	14 (27)	5 (24)	10 (29)	5 (33)	34 (41)	30 (60)
70 to 79 years	12 (27)	58 (28)	13 (25)	6 (29)	12 (34)	5 (33)	22 (27)	12 (24)
≥80 years	28 (62)	58 (28)	24 (47)	10 (48)	13 (37)	5 (33)	26 (32)	8 (16)
**Male sex n (%)**	28 (62)	110 (53)	23 (45)	15 (71)	24 (69)	10 (67)	43 (52)	23 (46)
World Health Organization severity ordinal scale [21] at admission n (%)****								
Ordinal scale 2	7 (16)	114 (55)	23 (45)	5 (24)	13 (37)	9 (60)	44 (54)	27 (54)
Ordinal scale 3	38 (84)	95 (45)	28 (55)	16 (76)	22 (63)	6 (40)	38 (46)	23 (46)
**Previous coexisting disease n (%)**								
Type 2 diabetes	15 (33)	58 (28)	15 (29)	8 (38)	9 (26)	4 (27)	27 (33)	10 (20)
Hypertension	28 (62)	124 (59)	34 (67)	12 (57)	25 (71)	8 (53)	45 (55)	28 (56)
Heart disease (excluding hypertension)	19 (42)	74 (35)	20 (39)	13 (62)	12 (34)	4 (27)	31 (38)	13 (26)
** **Chronic lung disease^¶^	8 (18)	51 (24)	12 (24)	5 (24)	7 (20)	4 (27)	20 (24)	11 (22)
** **Rheumatic diseases^+^	3 (7)	9 (4)	7 (14)	1 (5)	1 (3)	0 (0)	1 (1)	2 (4)
Cancer	7 (16)	22 (11)	7 (14)	5 (24)	2 (6)	2 (13)	7 (9)	6 (12)
Chronic kidney disease	6 (13)	9 (4)	4 (8)	3 (14)	4 (11)	1 (7)	2 (2)	1 (2)
**Number of coexisting diseases n (%)**								
None	3 (7)	38 (18)	4 (8)	0 (0)	5 (14)	3 (20)	15 (18)	14 (28)
One	18 (40)	65 (31)	18 (35)	6 (29)	12 (34)	6 (40)	24 (29)	17 (34)
Two or more	24 (53)	106 (51)	29 (57)	15 (71)	18 (51)	6 (40)	43 (52)	19 (38)
**Body mass index n (%)** ^ƒ^								
≥30.0	10 (22)	82 (39)	16 (31)	9 (43)	7 (20)	8 (53)	28 (34)	24 (48)
Missing data	20 (31)	14 (10)	9 (18)	1 (5)	8 (23)	1 (7)	13 (16)	2 (4)
**Treated with glucocorticoids n (%)** ^§^	24 (53)	134 (64)	29 (57)	14 (67)	23 (66)	7 (47)	46 (56)	39 (78)
Invasive mechanical ventilation n (%)***	8 (18)	7 (3)	7 (14)	0 (0)	3 (9)	0 (0)	3 (4)	2 (4)
**Died at 28 days n (%)^++++^**			23 (45)	8 (38)	7 (20)	3 (20)	3 (4)	1 (2)
**Median time (IQR) in days in hospital/to death or discharge^++++^**	9 (7)	8 (7)	8 (8)	10 (9)	13 (8)	12 (13)	7 (5)	6 (6)

^#^: recovered to hospital discharge – there was a significant difference between patients who had died and recovered (*^−^****), and/or clusters (^+–++++^), but there were no significant differences between the groups in any other characteristics; ^¶^ : chronic lung disease was defined as chronic obstructive pulmonary disease, asthma or chronic bronchitis; ^ƒ^: BMI is the weight in kilograms divided by the square of the height in metres; ^§^: the decision to prescribe medications was at the discretion of the treatment team for each patient. *p<0.05; **p<0.01; ***p<0.001; ****p<0.0001; Clusters C1–C6: **^+^**p<0.05; **^++^**p<0.01; **^+++^**p<0.001; **^++++^**p<0.0001.

#### Non-hospitalised control subjects

A group of 40 adult individuals with prior COVID-19 infection confirmed by a nasopharyngeal swab PCR test, but who were not hospitalised because of the disease, served as controls. In all control subjects, we collected a blood sample in a median of 60 days (range 20–175 days) after confirmation of SARS-CoV-2 infection. All control subjects provided informed consent. Data on demographics, including age and sex, are described in supplementary table E1 and in the online supplementary material.

The study protocol was approved by the Slovenian National Medical Ethics Committee (No. 0120-201/2020/7 and 0120-333/2020/3) and was registered at ClinicalTrials.gov (NCT04679428).

### Anti-SARS-CoV-2 spike 1 IgG antibodies, absolute quantitation of lymphocyte subsets and IL-6 measurements

IgG antibodies to SARS-CoV-2 anti-spike 1 were quantified using IDK anti-SARS-CoV-2 ELISA Kit (Immundiagnostik AG Bensheim, Germany) in a single serum sample per patient (254 samples) collected a median of 4 days after admission. Lymphocyte subsets were quantified in peripheral blood with EDTA using BD (Franklin Lakes, NJ, USA) Multitest™, a 6-colour direct immunofluorescence reagent, BD Trucount™ tubes and BD FACSCanto™ II flow cytometer with BD FACSCanto clinical software version 3.1 in 698 longitudinally collected blood samples. For all samples, we performed staining and flow cytometric analyses on the same day as blood venipuncture. Decreases in circulating blood lymphocyte subsets were defined as the lowest absolute counts recorded after admission to the hospital. IL-6 in serum samples was quantified using Immulite 2000 XPi (Siemens Healthcare GmbH, Erlangen, Germany) in 698 longitudinally collected serum samples. IL-6 increase was defined as the maximal serum level recorded after admission to the hospital. For all sampling from all patients, the timing of blood collection was in the morning, between 7:00 and 10:00 h.

In non-hospitalised control subjects, we quantified IgG antibodies to SARS-CoV-2 anti-spike 1, quantitative lymphocyte subsets and IL-6 from a single sample per subject.

### The outcome measure, regression and cluster analysis, and clinical association of the immune profiles

The primary outcome was mortality within 28 days after admission. All variables were analysed using Cox regression models in a stepwise manner – first univariate and then multivariate regression using only significant univariate predictors. Furthermore, we used a Gaussian mixture model [18] to cluster patients based on their antibody [2, 9], circulating lymphocyte subsets [3, 5, 14–17] and serum interleukin-6 [1, 3, 5, 8, 13] immune responses to SARS-CoV-2. Before clustering, all variables were normalised. The model was fitted by the expectation–maximisation algorithm, and the optimal number of clusters was chosen using the Bayesian Information Criterion. We then compared the primary outcome between different clusters using multivariable Cox proportional hazard models, adjusted for major clinical severity factors that were significant predictors of death in the above-mentioned regression analysis, and Kaplan–Meier survival curves were constructed to visualise mortality over the 28 days across clusters. Further details regarding data analysis are described in the online supplementary material. The analysis was carried out using R software [19, 20].

## Results

### Participant, demographic data and dynamic characterisation of anti-SARS-CoV-2 immune responses

The mean age of the patients was 71.8±13.9 years, and 138 (54.3%) were male. At admission, 133 (52.4%) were receiving oxygen (without invasive mechanical ventilation) – World Health Organization (WHO) severity ordinal scale 3; 121 (47.6%) were not receiving oxygen – WHO severity ordinal scale 2 [21]. A history of at least one major coexisting recorded illness was present in 213 (84%) patients. A total of 148 patients (58%) received dexamethasone [22], 10 (4%) received methylprednisolone [23] and 28 (11%) received remdesivir [24]. The decision to prescribe medications was at the discretion of the treatment team of each patient. During the study inclusion and patient follow-up (September–December 2020), there were no other medications available for the treatment of patients with COVID-19, and no vaccines were authorised in the European Union to prevent COVID-19. None of the patients was included in studies for approval of novel medications or vaccines.

During a follow-up of 3 to 26 days after admission, 45 patients (18%) died (eight subsequently after intubation). During a follow-up of 3 to 35 days after presentation, 209 patients had survived hospital discharge (seven after successful cessation of invasive mechanical ventilation). Six of those patients were discharged more than 28 days after the presentation (during a follow-up of 29 to 35 days; one after successful cessation of invasive mechanical ventilation). There was a significant age difference and difference in respiratory support at presentation (WHO severity ordinal scale) [21] between recovered patients and patients who had died; however, there were no significant differences in any other demographics or comorbidities (table 1).

Overall, patients who progressed to the primary end-point of death at 28 days showed a markedly lower level of anti-SARS-CoV-2 spike 1 IgG antibodies in comparison to patients recovered to hospital discharge (median (IQR) 381 (2258) *versus* 2701 (2080) pg·mL^−1^, p<0.0001; supplementary figure E1A). Patients who progressed to the primary end-point also showed significantly lower frequencies of anti-SARS-CoV-2 spike 1 IgG antibody positivity (62.2% *versus* 91.9% respectively: p<0.0001), according to the 175 ng·mL^−1^ positivity threshold (supplementary figure E1B).

Longitudinal immune profiling revealed that the patients who progressed to the primary end-point showed significant decreases in circulating T-cells and CD4 and CD8 subpopulations of T-cells, and an increase in serum IL-6 in comparison to patients who recovered to hospital discharge, with an overall median (IQR) cell count of 415 (310) *versus* 797 (630), 275 (225) *versus* 521 (422) and 119 (134) *versus* 229 (235) cells per μL, and median (IQR) IL-6 concentration of 59 (85) *versus* 13.8 (25.3) pg·mL^−1^ respectively; p<0.0001) in 698 samples (supplementary figure E2A–G). There was also a significant reduction for B-cell and natural killer (NK) cells (overall median 84 (101) *versus* 134 (135) and 95 (114) *versus* 145 (128) cells per μL; p<0.0001) (supplementary figure E2A,E and F). Furthermore, the differences (median (IQR)) in the lowest absolute counts or highest IL-6 between patients who progressed to the primary end-point and those who recovered to hospital discharge were 325 (239) *versus* 627 (506), 215 (179) *versus* 387 (330) and 100 (114) *versus* 188 (205) cells per μL for CD3 and CD4 and CD8 T-cells and 82.1 (138.7) *versus* 23.2 (44.2) pg·mL^−1^ for IL-6 (p<0.0001) (supplementary figure E3). Less marked differences were observed for the lowest B-cell and NK cell counts (68 (96) *versus* 101 (100), 94 (114) *versus* 122 (97), respectively; p≤0.04) (supplementary figure E3).

The mean±sd age of non-hospitalised control subjects was 45.6±12.1 years; there were 22 (55%) males (supplementary table E1). Results of comparison of the immunological parameters between non-hospitalised control subjects sampled a median of 60 days after SARS-CoV-2 infection and hospitalised recovered or deceased COVID-19 patients are shown in supplementary figures E1A-B, E2A and E3. The level and positivity of anti-SARS-CoV-2 spike 1 IgG antibodies were comparable between non-hospitalised control subjects and hospitalised patients that had recovered; however, they were significantly higher than in deceased hospitalised individuals (p<0.0001) (supplementary figure E1A–B). Furthermore, non-hospitalised control subjects had markedly higher CD3 T-, CD4 T-, CD8 T-, B- and NK cell counts and lower IL-6 levels than hospitalised recovered or deceased individuals (p<0.01) (supplementary figures E2A and E3).

### Predictors of fatal COVID-19

We tested the variables in univariate Cox regression models and early humoral response (anti-SARS-CoV-2 spike 1 IgG antibodies, hazard ratio (HR) 0.9993; 95% CI 0.9990–0.9996) was associated with the primary outcome (supplementary table E2). For circulating quantitative lymphocyte subsets there was a significant association for decreases (the lowest absolute count after admission) of T-cells (HR 0.9965; 95% CI 0.9951–0.9979), and CD4 (HR 0.9948; 95% CI 0.9926–0.9971; <500 CD4^+^ cells·mm^−3^: HR 8.9; 95% CI 2.16–36.8, and <200 CD4^+^ cells·mm^−3^: HR 3.8; 95% CI 2.13–6.86) and CD8 subpopulation of T-cells (HR, 0.9936; 95% CI, 0.9903 to 0.9969) and end-point of death. However, there was no association between B and NK cells decreases and death. (supplementary table E2). An increase in serum IL-6 (the maximal serum level recorded after admission) was also associated with the primary outcome (HR 1.0022; 95% CI 1.0012 to 1.0032). Comparable, as in previous studies [22, 24] older patients (HR 1.09; 95% CI 1.05–1.13) and those who received respiratory support (WHO severity ordinal scale 3) at the time of presentation (HR 5.7; 95% CI 2.55–12.77) were also more likely to have had a primary end-point event (supplementary table E2). However, there were no associations between the primary outcome, and coexisting diseases, BMI and glucocorticoid use.

Associations that were significant using univariate analysis were then estimated by multivariable Cox regression model. Using multivariable Cox model anti-SARS-CoV-2 spike 1 IgG antibodies (HR 0.9994; 95% CI 0.9991 to 0.9996), decreases in circulating T-cells (HR 0.9977; 95% CI 0.9963 to 0.9991) or CD4 (HR 0.9965; 95% CI 0.9943 to 0.9988) or CD8 (HR 0.996; 95% CI 0.9929 to 0.9991) subpopulations of T-cells and increase in IL6 (HR 1.0014; 95% CI 1.0001 to 1.0027) remained significant predictors of the primary end-point independent of older age (HR 1.0652; 95% CI 1.0294 to 1.1023) and WHO severity ordinal scale [21] at the time of presentation (HR 4.5264; 95% CI 1.9594 to 10.4563) (supplementary table E2).

### Profiles of anti-SARS-CoV-2 immune responses

**FIGURE 1:**
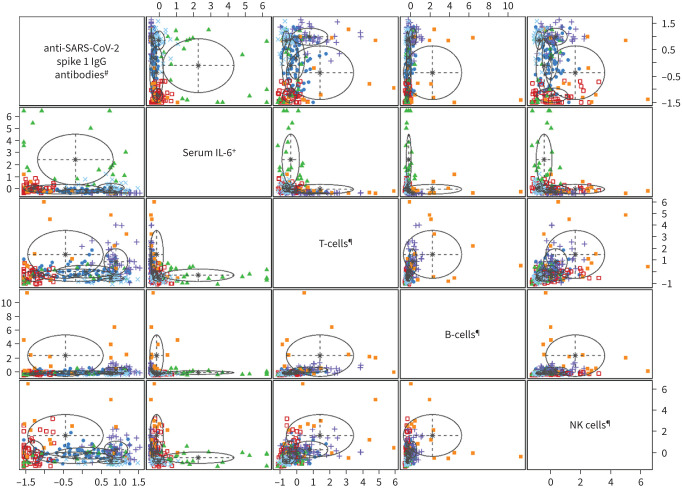
The visualization of Gaussian mixed model cluster plots. ^#^: SARS-CoV-2 anti-S1 IgG antibodies were measured a median of 4 days after admission to the hospital; ^¶^: decreases in circulating lymphocytes were defined as the lowest T-, B- and natural killer (NK) cell absolute counts recorded after admission; ^+^: interleukin-6 (IL-6) increase was defined as the maximal serum level recorded after admission.

**FIGURE 2:**
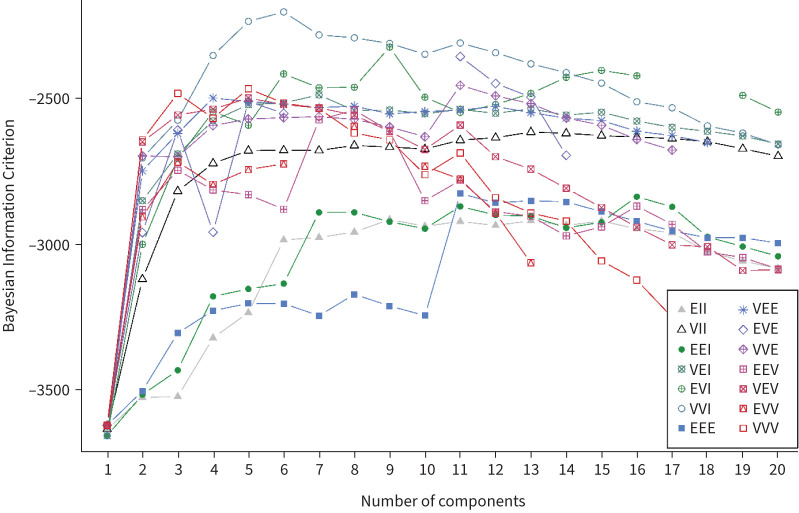
Determination of the optimal number of clusters with simultaneously run and comparison of several probabilistic models based on their Bayesian Information Criterion score. VVI model with the highest Bayesian Information Criterion score (optimum n=6) was preferred.

**FIGURE 3:**
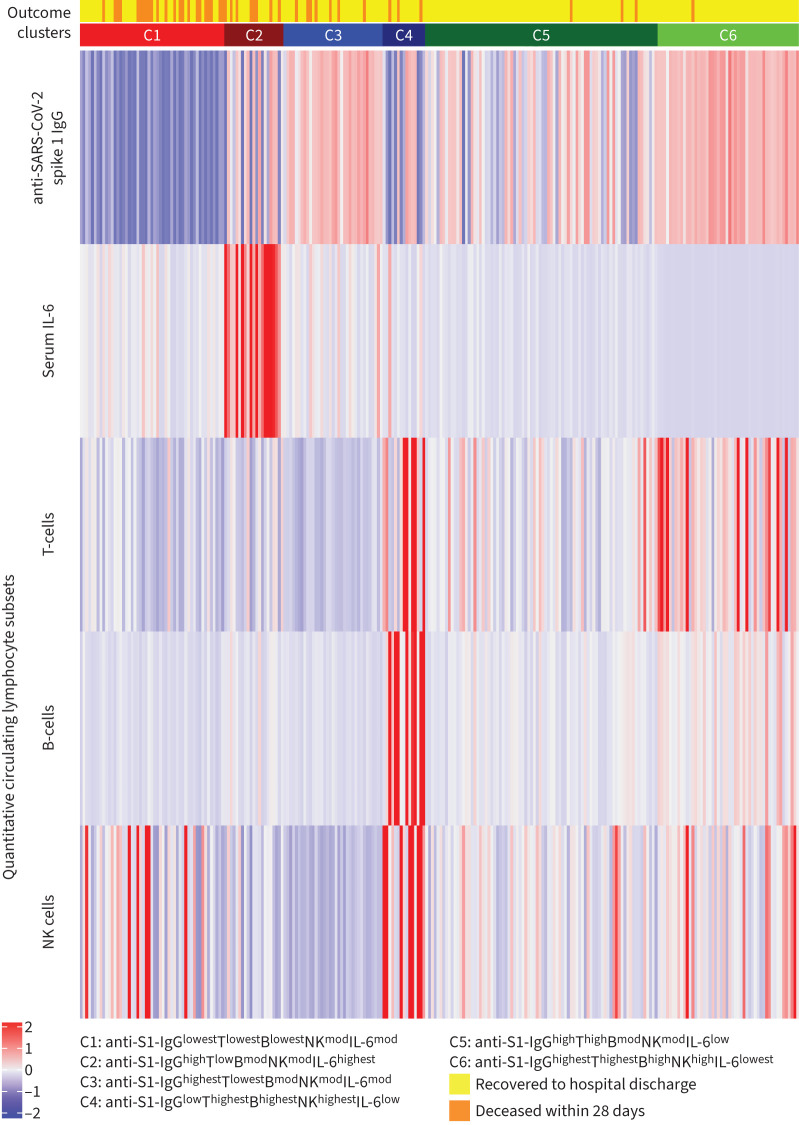
Immune responses of 254 hospitalised COVID-19 patients across the six clusters (C1–6). The normalised values of parameters are shown. Heatmap: red indicates the individual response is greater than the population mean, and blue indicates that the individual response is less than the population mean. SARS-CoV-2 anti-S1 IgG antibodies were measured a median of 4 days after admission to the hospital. Decreases in circulating lymphocyte subsets were defined as the lowest T-cell, B-cell and natural killer (NK) cell absolute counts recorded after admission. Interleukin-6 (IL-6) increase was defined as the maximal serum level recorded after admission.

A six-cluster model provided an optimal solution of the Gaussian mixed model algorithm (VVI) according to the Bayesian Information Criterion statistic (Figures 1 and 2). Figure 3 shows the individual patterns of anti-SARS-CoV-2 spike 1 IgG antibodies, circulating T-cell, B-cell, NK cell counts and serum IL-6 across the six clusters in terms of the normalised value of each parameter. We assigned the relative expression in clusters as “highest”, “high”, “moderate“, “low” or “lowest” (figure 3 and table 2). Cluster 1 (C1, n=51, 20.1%) was characterised by the lowest anti-SARS-CoV-2 spike 1 IgG antibodies and T- and B-cell counts, and moderate NK cells and IL-6 level (anti-S1-IgG^lowest^T^lowest^B^lowest^NK^mod^IL-6^mod^). Cluster 2 (C2, n=21, 8.3%) was characterised by the highest IL-6 level, high anti-S1-IgG, low T-cells, and moderate NK and B-cells (anti-S1-IgG^high^T^low^B^mod^NK^mod^IL-6^highest^). Patients in cluster 3 (C3, n=35, 13.8%) had the highest anti-S1-IgG and the lowest T-cell counts, moderate B- and NK cells and IL-6 level (anti-S1-IgG^highest^T^lowest^B^mod^NK^mod^IL-6^mod^). Cluster 4 was the smallest (C4, n=15, 5.9%) and was characterised by low anti-S1 IgG and IL-6 and the highest T-, B- and NK cells (anti-S1-IgG^low^T^highest^B^highest^NK^highest^IL-6^low^). Patients in cluster 5 (C5, n=82, 32.3%) and cluster 6 (C6, n=50, 19.7%) had high or the highest anti-S1 IgG and T-cells, low or the lowest IL-6 and high or moderate B- and NK cells (anti-S1-IgG^high^T^high^B^mod^NK^mod^IL-6^low^ and anti-S1-IgG^highest^T^highest^B^high^NK^high^IL-6^lowest^, respectively).

**TABLE 2:** Antibody response, lymphocytes and interleukin-6 (IL-6) according to cluster assignment

	**Cluster 1**	**Cluster 2**	**Cluster 3**	**Cluster 4**	**Cluster 5**	**Cluster 6**
**Subjects n (%)**	51 (20.1)	21 (8.3)	35 (13.8)	15 (5.9)	82 (32.3)	50 (19.7)
**Anti-SARS-CoV-2 S1 IgG antibodies pg·mL^−1^** ^#^						
Minimum	0	0	1425	0	0	1958
25%	0	301.9	2850	184.7	1317	2934
Median	**191.7**	**2246.2**	**3013**	**877.1**	**2113**	**3074**
75%	450	2969.7	3152	2923.2	2848	3286
Maximum	1019.6	3423.5	3828	3444.7	3556	3893
**T-cells per mm^3^** ^¶^						
Minimum	95.0	88.0	93.0	227	191.0	159.0
25%	252.0	338.0	266.5	594	489.2	729.0
Median	**372.0**	**418.0**	**343.0**	**1015**	**619.0**	**954.5**
75%	644.0	767.0	422.0	2010	743.2	1208.2
Maximum	1008.0	992.0	557.0	3662	1574.0	2650.0
**B-cells per mm^3^** ^¶^						
Minimum	0.00	16.0	13.00	71.0	16.00	31.0
25%	40.50	50.0	47.50	214.0	72.25	119.2
Median	**56.00**	**101.0**	**67.00**	**660.0**	**103.00**	**188.0**
75%	83.00	129.0	89.50	973.0	147.00	262.0
Maximum	161.00	271.0	121.00	3097.0	286.00	467.0
**NK cells per mm^3^** ^¶^						
Minimum	7.0	7.00	20.00	83.0	31.0	42.0
25%	72.5	49.00	38.00	135.0	92.0	110.5
Median	**113.0**	**103.00**	**56.00**	**259.0**	**125.0**	**146.0**
75%	185.5	131.00	69.50	399.5	162.8	202.5
Maximum	459.0	201.00	106.00	790.0	327.0	362.0
**IL-6 pg·mL^−1^** ^+^						
Minimum	4.93	93.2	8.14	3.48	2.96	2.00
25%	34.45	2018.0	24.90	16.55	16.02	3.692
Median	**57.70**	**315.0**	**58.20**	**32.10**	**24.85**	**5.34**
75%	89.55	620.0	105.00	59.35	40.35	7.28
Maximum	184.0	1000.0	237.00	232.00	71.50	13.10

^#^: SARS-CoV-2 anti-S1 IgG antibodies were measured a median of 4 days after admission to the hospital; ^¶^: decreases in circulating lymphocytes were defined as the lowest T-, B- and natural killer (NK) cell absolute counts recorded after admission; ^+^: IL-6 increase was defined as the maximal serum level recorded after admission.

### Characteristics of anti-SARS-CoV-2 immune response clusters

#### Demographic, baseline and treatment features

Patients in C1, C2 and C3 were older (mean age: 75.3, 77 and 74.2 years, respectively) than patients in C4, C5 and C6 (mean age: 71.8, 70 and 67.5 years, respectively; p=0.003) (table 1). Patients in C1 (14%) had more rheumatic disease than patients in C2, C3, C4, C5 and C6 (5%, 3%, 0%, 1% and 4%, respectively; p=0.03). Patients in C3 and C4 stayed longer in the hospital to discharge or death (median 13 and 12 days, respectively) than patients in C1, C2, C5 and C6 (median 8, 10, 7 and 6 days, respectively, p<0.0001)*.* There was no significant association between cluster membership and sex, WHO severity ordinal scale at admission [21], previous coexisting disease including type 2 diabetes, hypertension and heart diseases, chronic lung disease, cancer, chronic kidney disease, and the number of coexisting diseases, body mass index and corticosteroid treatment (table 1).

#### Fatal COVID-19

To test if anti-SARS-CoV-2 immune response clusters identified by employing unsupervised machine learning are associated with the risk of fatal COVID-19 independent of clinical severity factors we constructed a grid of Cox regression models. Thus we used age and WHO severity ordinal scale [21] at admission, which were the only non-immunological predictors of mortality in multivariable Cox regression (supplementary table E2), together with cluster status as predictor variables. Results of the regression models to investigate fatal COVID-19 outcomes in various clusters are shown in tables 3 and 4. Patients in the highest-risk C1 cluster (anti-S1-IgG^lowest^T^lowest^B^lowest^NK^mod^IL-6^mod^) had 3.36, 15.34 and 21.69 times higher hazard of death due to COVID-19 (95% CI 1.51–8.55 and 4.55–51.72; 2.89–163.61; P≤0.004) than patients in C3, C5 and C6, respectively. Patients in high-risk C2 (anti-S1-IgG^high^T^low^B^mod^NK^mod^IL-6^highest^) had 8.39 and 10.97 times higher hazard of death due to COVID-19 (95% CI, 2.17–32.51 and 1.20–82.67; P≤0.03) than patients in C5 and C6, respectively. Patients in moderate-risk C3 (anti-S1-IgG^highest^T^lowest^B^mod^NK^mod^IL-6^mod^) had 5.24 times higher hazard of death due to COVID-19 (95% CI 1.34–20.48 p=0.02) than patients in C5. Similarly, patients in moderate-risk C4 (anti-S1-IgG^low^T^highest^B^highest^NK^highest^IL-6^low^) cluster had 5.36 times higher hazard of death due to COVID-19 than patients in C5 cluster (95% CI 1.08–26.67; p=0.04). When comparing moderate-risk clusters C3 and C4 to cluster C6, the hazards of dying from COVID-19 were higher; however, they failed (due to small sizes) to reach statistical significance. Patients in C5 (anti-S1-IgG^high^T^high^B^mod^NK^mod^IL-6^low^) and C6 (anti-S1-IgG^highest^T^highest^B^high^NK^high^IL-6^lowest^) were at very low risk of fatal COVID-19, and this very low risk was comparable between the two clusters (HR 1.66; 95% CI 0.17–16.43; p=0.67). Since the similar risks of fatal COVID-19 between clusters C1 and C2, clusters C3 and C4, clusters C5 and C6, and since some clusters were small (C2: N=21, 8.3%, and C4: N=15, 5.9%) regression analysis was also performed comparing grouped clusters C1+C2, C3+C4, and C5+C6) (table 4). Patients in clusters C1+C2 had 2.67 and 13.73 times higher hazard of death due to COVID-19 (95% CI 1.30–5.48 and 4.79–39.36; p≤0.008) than patients in clusters C3+C4 and C5+C6, respectively. Furthermore, patients in clusters C3+C4 had 5.90 times higher hazard of death due to COVID-19 (95% CI 1.84–18.97; p=0.003) than patients in clusters C5+C6.

**TABLE 3:** Risk of fatal COVID-19 in various clusters

	**Cluster 1**	**Cluster 2**	**Cluster 3**	**Cluster 4**	**Cluster 5**	**Cluster 6**
**Subjects n (%)**	51 (20.1)	21 (8.3)	35 (13.8)	15 (5.9)	82 (32.3)	50 (19.7)
**Cluster 1**						
**Cluster 2**	1.94 (0.86–4.37), p=0.11					
**Cluster 3**	**3.36 (1.51–8.55), p=0.004**	1.87 (0.66–5.24), p=0.24				
**Cluster 4**	2.95 (0.88–9.93), p=0.08	1.51 (0.39–5.79), p=0.55	0.88 (0.23–3.41), p=0.89			
**Cluster 5**	**15.34 (4.55–51.72), p<0.0001**	**8.39 (2.17–32.51), p=0.002**	**5.24 (1.34–20.48), p=0.02**	**5.36 (1.08–26.67), p=0.04**		
**Cluster 6**	**21.69 (2.89–163.61), p=0.003**	**10.97 (1.20–82.67), p=0.03**	7.33 (0.89–60.24), p=0.06**7**	7.41 (0.75–72.75), p=0.09	1.66 (0.17–16.43), p=0.67	

Data presented as HR (95% CI), p-value unless otherwise indicated. Values represent hazard ratios for death from COVID-19, with 95% confidence intervals for the average effect of each cluster compared with other clusters. p-values assess whether the distribution of characteristics within a given cluster is significantly different from a characteristic in other clusters in a regression model adjusted for age and WHO severity ordinal scale [21] at the time of admission. Numbers highlighted in boldface represent clusters that had significantly (p<0.05) different characteristics from characteristics in other clusters. CI: confidence interval; HR: hazard ratio; WHO: World Health Organization.

**TABLE 4:** Risk of fatal COVID-19 in grouped clusters

	**Clusters 1+2 (N=72, 28.4%)**	**Clusters 3+4 (N=50, 19.7%)**	**Clusters 5+6 (N=132, 52%)**
**Subjects n (%)**	72 (28.4)	50 (19.7)	132 (52)
**Clusters 1+2**			
**Clusters 3+4**	**2.67 (1.30–5.48), p=0.008**		
**Cluster 5+6**	**13.73 (4.79–39.36), p<0.0001**	**5.90 (1.84–18.97), p=0.003**	

Data presented as HR (95% CI), p-value unless otherwise indicated. Values represent hazard ratios for death from COVID-19, with 95% confidence intervals for the average effect of each grouped clusters compared with other grouped clusters. p-values assess whether the distribution of characteristics within a given cluster is significantly different from a characteristic in other clusters in a regression model adjusted for age and WHO severity ordinal scale [21] at the time of admission. Numbers highlighted in boldface represent clusters that had significantly (p<0.05) different characteristics from characteristics in other clusters. CI: confidence interval; HR: hazard ratio; WHO: World Health Organization.

**FIGURE 4:**
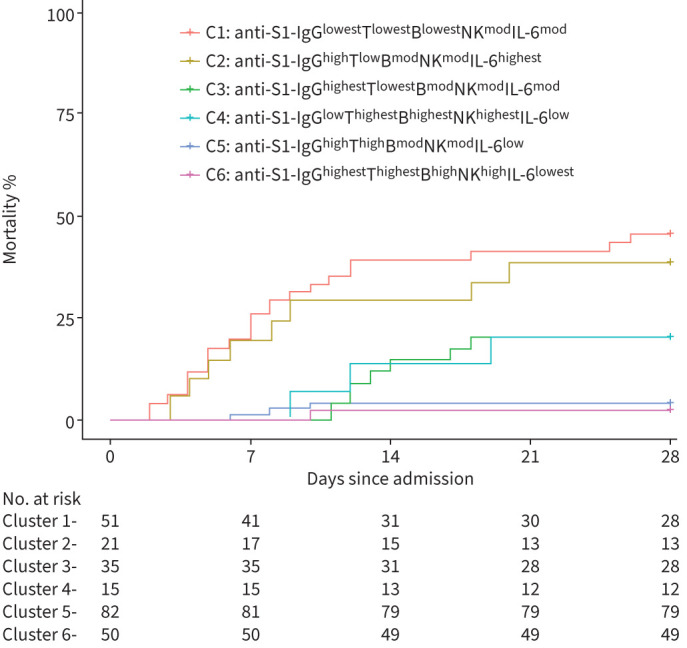
Kaplan–Meier survival curves for 28-day mortality according to the membership of the six clusters.

Kaplan–Meier estimates of mortality by day 28 were high, with more than 45% mortality in C1 (45.1%; 95% CI 29.6–57.2%) and with almost 40% mortality in C2 (38.1%; 95% CI 19–56.7%), and moderate with 20% mortality (half the mortality of C1 and C2) in C3 (95% CI 5.6–32.2%) and C4 (95% CI 0–37.9%). In contrast, Kaplan–Meier estimates of mortality were very low in C5 and C6 (10 to 23 times lower as in C1 and C2, 5 to 10 times lower as in C3 and C4) with 3.7% mortality in C5 (95% CI 0–7.6%) and 2% mortality in C6 (95% CI 0–5.8%) (figure 4).

## Discussion

By employing unsupervised machine-learning approaches, we identified discrete immunophenotypes of anti-SARS-CoV-2 responses represented by six clusters, which differed considerably in COVID-19 survival. Two clusters were associated with a high risk of fatal COVID-19 (38–45% mortality) and were defined as the lowest humoral response with the lowest circulating T-cells (C1), or as high IL-6 inflammatory response (C2). In contrast, two clusters (C5 and C6) were associated with a very low risk (2–4% mortality) and were defined as high or highest humoral and T-cells, with low or lowest IL-6. We also identified two clusters (C3 and C4) with moderate risk (20% mortality) characterised by unfavourable low humoral (C4) or lowest T-cells (C3), but with favourable highest T-cells (C4) and/or low (C4) or moderate (C3) IL-6 responses.

First studies suggested that anti-SARS-CoV-2 antibodies are higher in patients with severe disease [25]. However, this association probably only reflected the more protracted disease course of the severe disease. In contrast, further longitudinal and larger studies [26–28] have shown that higher levels of antibodies targeting the spike protein (as opposed to targeting the nucleocapsid protein) if present early in disease development are predictors of favourable disease outcome and that the lack of humoral response is associated with deleterious disease course. This is probably because early humoral response helps in constraining the viral infection [27], preventing it from spreading to other parts of the body, and thus restricts the escalation of disease severity. As seen by our results and also by other recent studies [26–28], impaired or delayed antiviral humoral immunity might be one of the strongest risk factors for fatal COVID-19; in fact, in our cohort, the biggest proportion (more than 50%) of deceased patients was attributable to the C1 in which individuals showed the lowest humoral response (5- to 16-fold lower as in other clusters), measured in a time frame of a few days after admission. Therefore, we speculate that at hospitalisation, antiviral antibody status should be evaluated in all patients, not only for risk-based stratification but also to possibly identify individuals in which prompt antibody treatment may reduce the risk of disease progression [29]. This humoral response-based risk stratification/treatment venue may become even more critical with future variants of concern when patients would be vaccinated against or will recover from the disease caused by one variant but get infected by another significantly different variant. In such cases, it would be important to evaluate the antiviral antibody status specifically for the acute disease-causing variant and thus possibly choose the most efficient antibody treatment available (*i.e.* specific monoclonal antibodies and/or variant-specific convalescent plasma).

The other high-risk cluster (C2) was smaller and characterised by hyper-inflammation as measured by the highest IL-6 levels. In this cluster, IL-6 levels were 5- to 60-fold higher than in other clusters. Systemic hyper-inflammation with elevated levels of IL-6, sometimes referred to as the “cytokine storm”, is a well-recognised phenomenon of severe COVID-19 [30–33]. Importantly, patients in this high-risk hyper-inflammatory cluster showed a protective high antiviral humoral response; conversely, patients in high-risk C1 with impaired humoral viral control showed low-risk moderate IL-6 levels. These findings suggest that there may be two discrete immune pathways leading to disease progression and fatal COVID-19. One may be driven by delayed kinetics of antibody production, independently of hyper-inflammation, and the other may arise through excessive IL-6-mediated host inflammation response and independently of the protective humoral response. As opposed to patients with delayed kinetics of antibody production, but no hyper-inflammation, which should be treated by providing them with humoral immune support (antibody treatment or convalescent plasma), the patients with hyper-inflammation (as reflected by high IL-6 levels), but sufficient humoral response, should be treated by immune suppression (*i.e.*, anti-IL6 and/or glucocorticosteroids). Our data therefore suggest the possible clinical importance of anti-SARS-CoV-2 immunophenotyping for the possible selection of more efficient personalised antiviral therapies.

Lymphocytes, especially T-cells, are key orchestrators of antiviral immune responses [10]. However, the contribution of T-cells to anti-SARS-CoV-2 protective or host pathogenic responses remains poorly understood. An imbalance of SARS-CoV-2-reactive CD4^+^ T-cells, with increased proportions of cytotoxic T-helper cells and reduced proportions of Treg cells [4], and T-cell chemoattractants and enrichment of interferon gamma-producing T-cells in the alveolar space [11], suggests the pathogenic role of T-cells. On the other hand, T-cell lymphopenia in lung [14] and impaired T-cell responses with no increase in T-cell abundances in the lungs have been reported in COVID-19 decedents [34]. From our data, we speculate that a significant decrease in circulating T-cells may be a surrogate marker of T-cell host pathogenic response and consequently, it was predominant in high-risk C1 and C2, and medium-risk C3 clusters. In contrast low or no decrease in circulating T-cells may be a surrogate marker of protective T-cell immune response, as it was predominant in moderate C4 or very low-risk clusters C5 and C6.

There are some important limitations to our study. The first and the most significant limitation is that we do not have a validation cohort. Validation in machine learning is relevant for assuring accuracy of the trained algorithm. However, acquisition of such large amounts of quality clinical and biological data is difficult and, to our knowledge, the only study that did such a comprehensive evaluation of COVID-19 patients and offers datasets freely available, the COVID-IP cohort, lacks the primary outcome strength that was present in our cohort. In the COVID-IP cohort [31] only five individuals had died; however in our cohort, we have recorded 45 cases of fatal COVID-19. Another limitation of the study is that clusters were not balanced concerning different baseline parameters, some of which (*i.e.* age) are also connected to the risk of death and thus might have confounded the observed results. However, the association between immunological clusters and the risk of death preserved its statistical significance also after major clinical risk factors (age, WHO severity ordinal scale category) were adjusted for in multivariable Cox logistic regression analysis. Moreover, due to the observational nature of the study, there were differences in the time at which samples were collected. However, anti-spike 1-IgG antibody levels were measured uniformly and early during the disease progression (median 4th day after the admission), and the kinetics of IL6 and circulating T-cells were monitored longitudinally during the disease course. Furthermore, circadian rhythm sensitive parameters (*i.e.* quantitative lymphocyte subsets) were measured at appropriate and consistent time-points.

In conclusion, by employing unsupervised machine learning we identified multiple anti-SARS-CoV-2 immune response profiles and observed major differences in COVID-19 mortality between these profiles. Our findings suggest that there may be different immune mechanisms that govern COVID-19 disease outcomes, and those observations could be explored further for application in clinical practice, possibly by immune monitoring and biomarkers for selecting novel drugs and existing COVID-19 treatments.

## Supplementary material

10.1183/23120541.00216-2022.Supp1 **Please note:** supplementary material is not edited by the Editorial Office, and is uploaded as it has been supplied by the author.
Supplementary material 00216-2022.supplement
