# Supplementary material for: Immunophenotypes of anti-SARS-CoV-2 responses associated with fatal COVID-19
Source: ERJ Open Res. 2022 Dec 5;8(4):00216-2022. doi: 10.1183/23120541.00216-2022 (PMC9510901; doi:10.1183/23120541.00216-2022)
Supplement: Supplementary file 1 [file 00216-2022.supplement.pdf]

Immunophenotypes of anti-SARS-CoV-2 responses and fatal COVID-19

ONLINE DATA SUPPLEMENT

Julij Šelb, M.D., Ph.D.[1*], Barbara Bitežnik, M.D.[1,2*], Urška Bidovec Stojković, Ph.D.[1], Boštjan Rituper, M.D., Ph.D.[1,2], Katarina Osolnik, M.D.[1], Peter Kopač, M.D.[1,2], Petra Svetina, M.D.[1], Kristina Cerk Porenta M.D.[1], Franc Šifrer M.D.[1], Petra Lorber M.D.[1,2], Darinka Trinkaus Leiler M.D.[1], Tomaž Hafner M.D[1,2], Tina Jerič M.D.[1], Robert Marčun M.D., Ph.D.[1,2], Nika Lalek M.D., Ph.D.[1,2], Nina Frelih M.D.[1], Mojca Bizjak M.D.[1], Rok Lombar M.D.[1], Vesna Nikolić M.D.[1,2], Katja Adamič M.D.[1], Katja Mohorčič M.D.[1], Sanja Grm Zupan M.D.[1], Irena Šarc, M.D.[1,2], Jerneja Debeljak MSc[1], Ana Koren, Ph.D. [1], Ajda Demšar Luzar MSc[1], Matija Rijavec, Ph.D.[1,3], Izidor Kern M.D.[1], Matjaž Fležar M.D., Ph.D. [1,2], Aleš Rozman, M.D., Ph.D.[1,2], and Peter Korošec, Ph.D.[1,4]

[1]University Clinic of Respiratory and Allergic Diseases, Golnik, Slovenia; [2]Medical Faculty, University of Ljubljana, Ljubljana, Slovenia; [3]Biotechnical Faculty, University of Ljubljana, Ljubljana, Slovenia;[4]Faculty of Pharmacy, University of Ljubljana, Ljubljana, Slovenia

*These authors contributed equally.

Correspondence: Peter Korošec Ph.D.; University Clinic of Respiratory and Allergic Diseases Golnik; Golnik 36, 4204 Golnik, Slovenia; E-mail: peter.korosec@klinika-golnik.si

Study participants.

We conducted this study of COVID-19 at the University Clinic of Respiratory and Allergic Diseases Golnik, a quaternary, acute care hospital in Slovenia. The first hospitalized patient was included on September 4th and the last on December 12th, 2020. All information presented in this report is based on a data cut-off of January 9th, 2021. During a follow-up of 3 to 26 days after presentation, 45 patients (18%) died (8 after intubation), with December 16th, 2020, as the time of the last endpoint event. During a follow-up of 3 to 35 days after presentation, 209 patients had survived hospital discharge (7 after successful cessation of invasive mechanical ventilation), with December 17, 2020, as the time of discharge of the last patients. Six of those patients were discharged more than 28 days after the presentation (during a follow-up of 29 to 35 days; one after successful cessation of invasive mechanical ventilation). None of the patients was transferred to anothe

For non-hospialized control subjects we included 40 adult individuals with prior COVID-19 confirmed by a nasopharyngeal swab PCR test (Supplement Table E1). In first control subject SARS-CoV-2 infection was confirmed on April 19th, 2021 and in the last on February 18th, 2022. The first blood sampling was done on July 12th, 2021 and the last on March 16th, 2022. Consequently a single post-blood sample was collected median of 60 days (range 20 to 175 days) after infection. None of the controls subjects was vaccinated neither before infection nor before sampling.

Data analysis

First, we performed a standard Cox proportional hazards analysis. The analysis was done with all the variables and in a stepwise manner. We analysed the variables in an univariate fashion and if the variable correlated significantly with the risk of death in univariate analysis it was further used as predictor in multivariable analysis (Supplement Table E1).

Regression analysis (including Cox regression) is considered a supervised machine learning method, since it fits the predictors to the outcome of interest (i.e. fatal COVID-19). To search for patterns in immune response that are not fitted to the outcome of interest and thus possibly reflect inherent biological processes, we next performed cluster analysis (unsupervised machine learning method) of immune variables. We used Gaussian mixture model algorithm as previously described[1]. Clustering was performed with variables of anti-SARS CoV-2 immune respose for which several studies demonstrated that likely contribute to disease severity. Those variables included antibody[2,3], circulating lymphocyte subsets[4–9] and serum interleukin-6[1,3,5,8,13] responses. Before clustering, all variables were normalized. The model was fitted by the expectation-maximization algorithm and the optimal number of clusters was chosen using the Bayesian information criterion (global optimum n=6 clusters; Figure 1).

We compared the end point characteristics of each cluster with other clusters, and Kaplan–Meier survival curves were constructed to visualize mortality over the 28 days across clusters. To account for possible differences between clusters in potential cofounding variables (i.e. clinical severity factors[14,15] that could influence the risk of fatal COVID-19), we used multivariable Cox regression with clusters and clinical severity factors serving as predictors of fatal COVID-19. We included only the non-immunological factors that were significant predictors of the fatal COVID-19 in a before constructed (please see above) multivariable adjusted Cox proportional hazard model.

Figure E1A-B. Anti-SARS-CoV-2 spike 1 IgG antibodies levels in 254 hospitalized COVID-19 patients according to whether patients progressed to the primary endpoint of death at 28 days or recovered to hospital discharge. **A** shows individual concentrations and **B** positivity of antibody testing. Non-hospitalized controls represent adult individuals with prior COVID-19 confirmed by a nasopharyngeal swab PCR test, post-sampling median 60 days after infection.

The median time of single blood sampling for both (hospitalized) subgroups was 4 days since hospital admission. A dotted horizontal line represents the cut-off between positive and negative antibody concentrations, assigned according to the manufacturer threshold for positivity (175 ng/ml). Horizontal lines represent the median with IQR. **** P<0.0001.

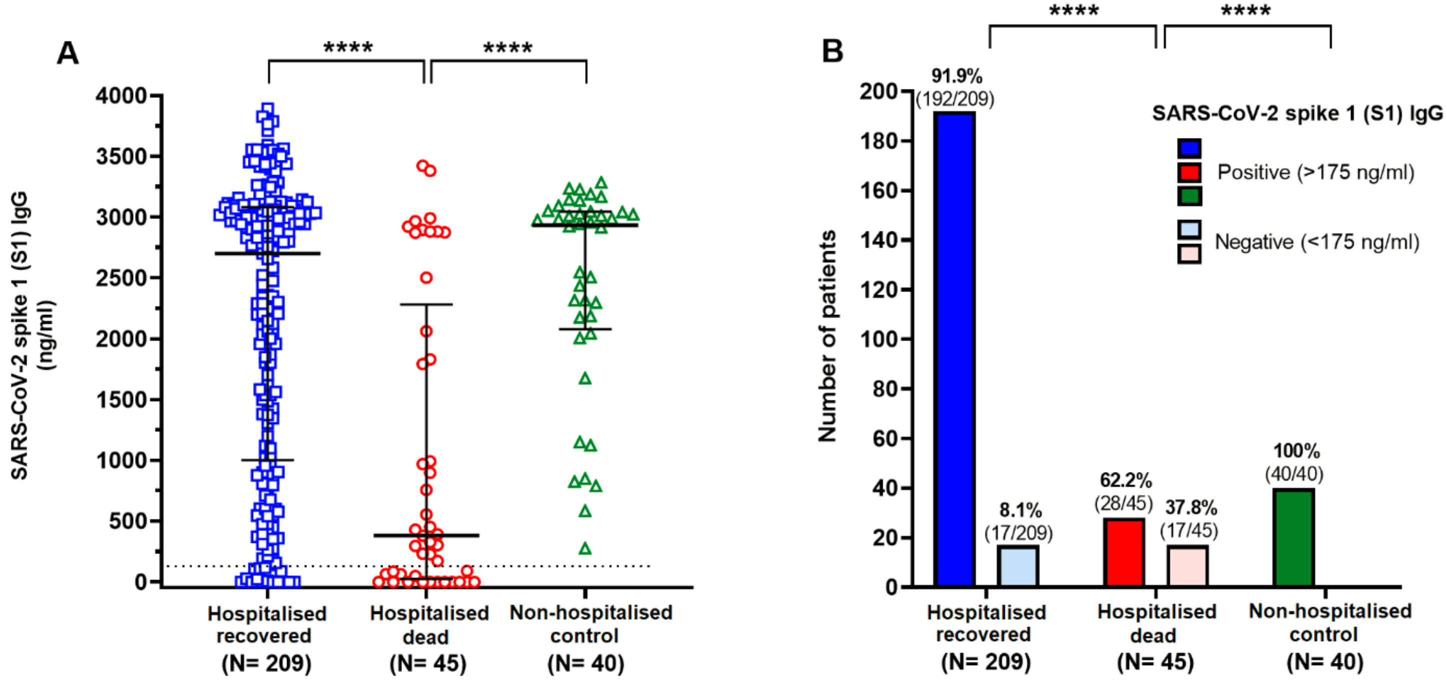

Figures E2A-G. A shows overall absolute counts of circulating T cells, CD4 and CD8 subpopulations of T cells, B and NK cells, and IL-6 measurements in sera, and longitudinal dynamic of circulating **B** T cells, **C** CD4 and **D** CD8 subpopulations of T cells, **E** B and **F** NK cell and **G** IL-6 in sera of hospitalized COVID-19 patients per day after admission according to whether patients progressed to the primary endpoint of death at 28 days or recovered to hospital discharge. Non-hospitalized controls represent adult individuals with prior COVID-19 confirmed by a nasopharyngeal swab PCR test, post-sampling median 60 days after infection.

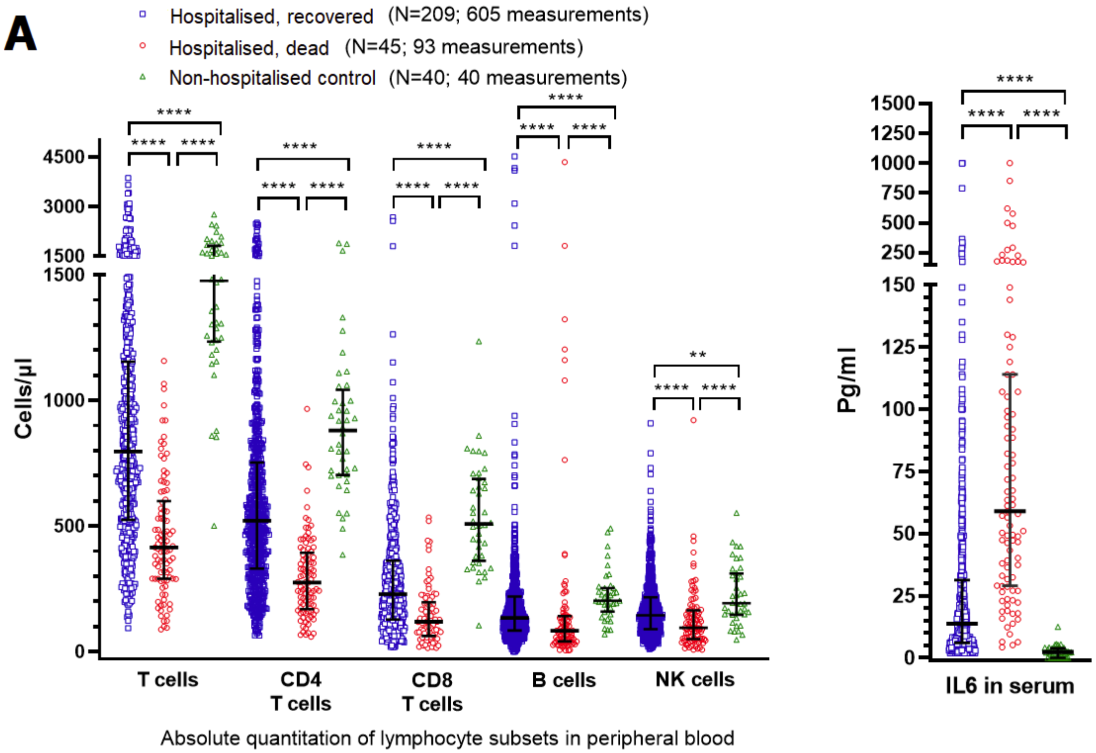

Data are shown as median with interquartile ranges continuously per day. Dotted or dashed horizontal lines are showing reference intervals. * P<0.05; ** P<0.01; *** P<0.001, and **** P<0.0001.

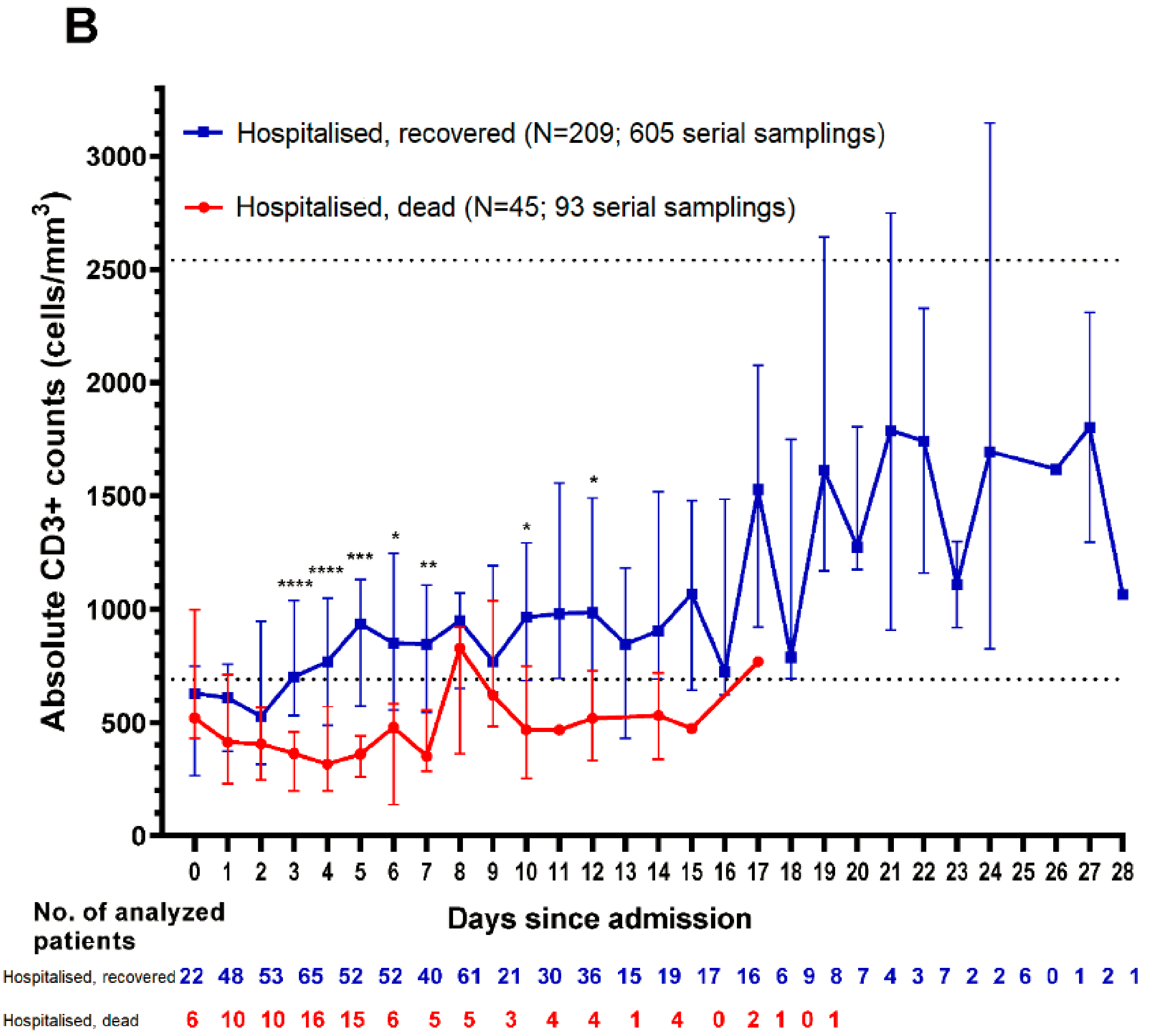

C

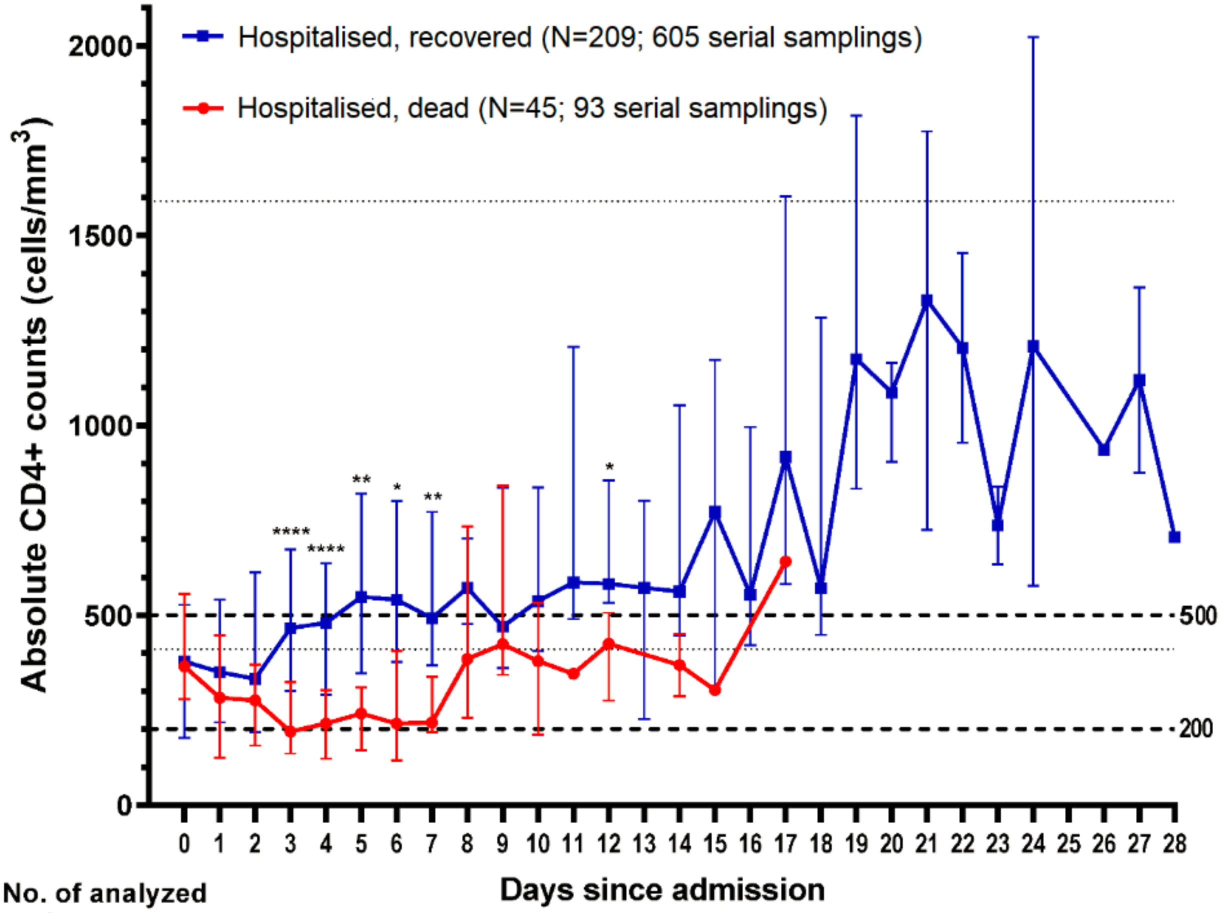

No. of analyzed patients

Hospitalised, recovered	22	48	53	65	52	52	40	61	21	30	36	15	19	17	16	6	9	8	7	4	3	7	2	2	6	0	1	2	1
Hospitalised, dead	6	10	10	16	15	6	5	5	3	4	4	1	4	0	2	1	0	1											

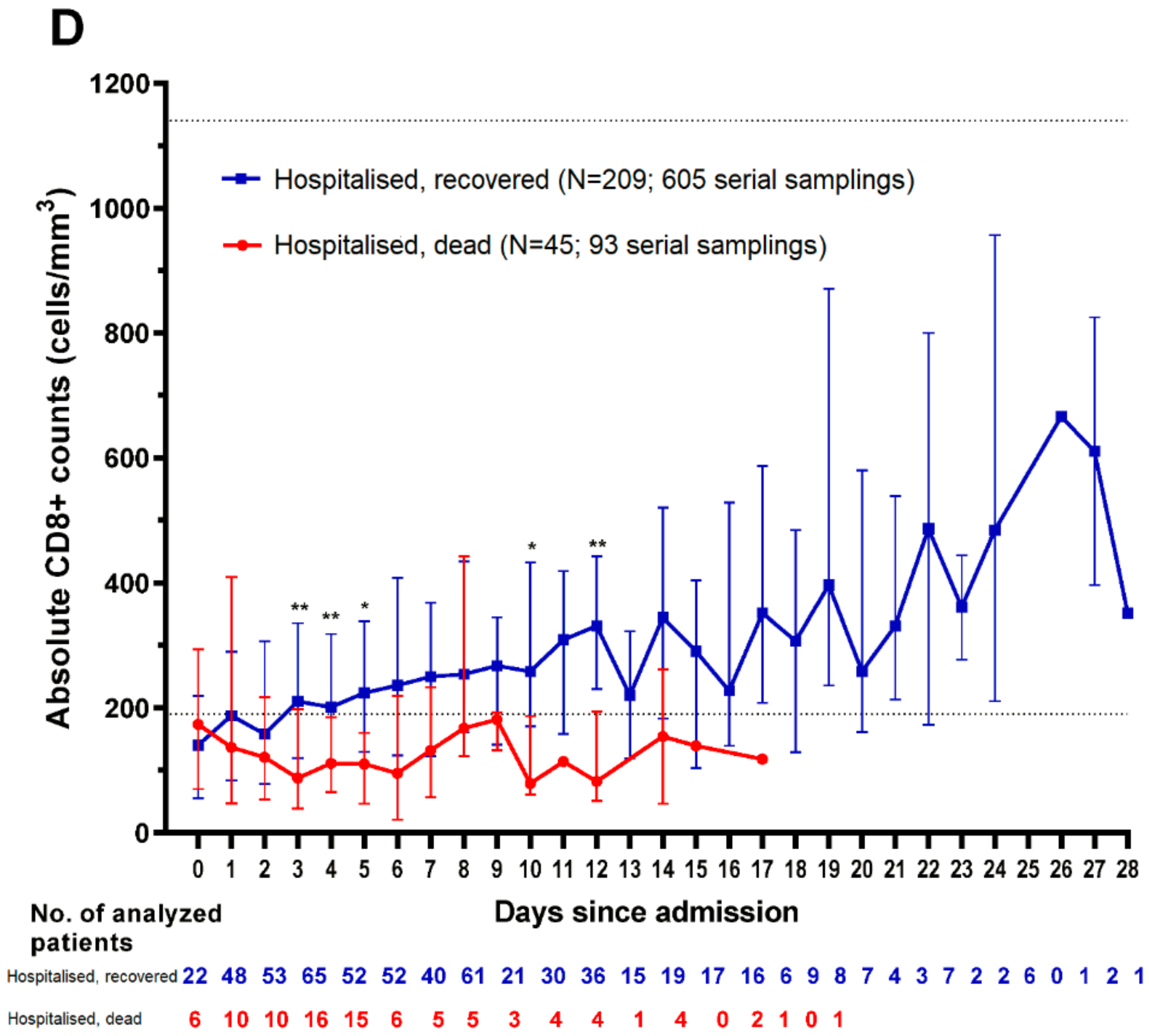
D
Absolute CD8+ counts (cells/mm3)
1200
1000
800
600
400
200
0
Hospitalised, recovered (N=209; 605 serial samplings)
Hospitalised, dead (N=45; 93 serial samplings)
** ** * * **
0 1 2 3 4 5 6 7 8 9 10 11 12 13 14 15 16 17 18 19 20 21 22 23 24 25 26 27 28
Days since admission
No. of analyzed patients
Hospitalised, recovered 22 48 53 65 52 52 40 61 21 30 36 15 19 17 16 6 9 8 7 4 3 7 2 2 6 0 1 2 1
Hospitalised, dead 6 10 10 16 15 6 5 5 3 4 4 1 4 0 2 1 0 1

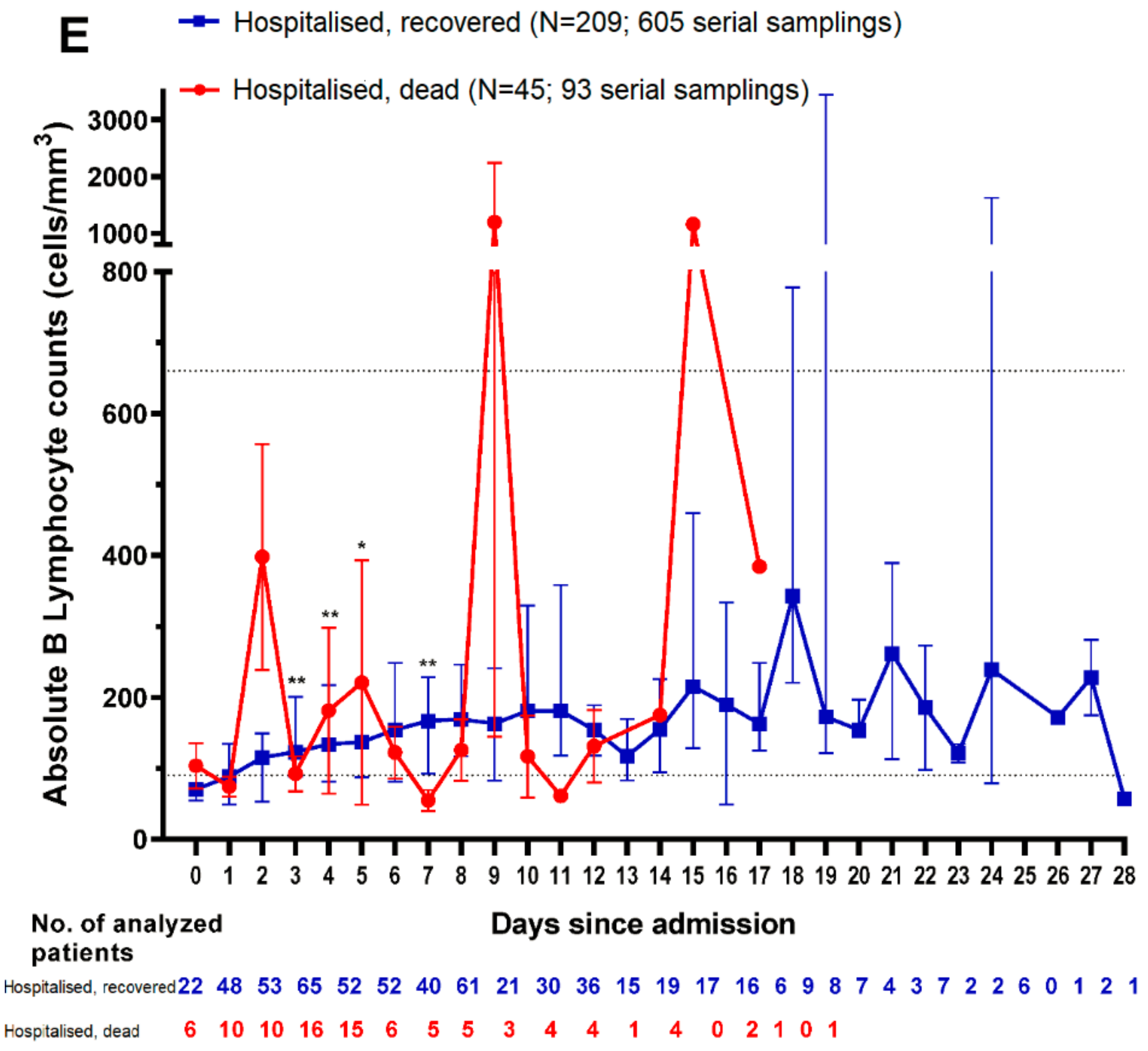
E
Hospitalised, recovered (N=209; 605 serial samplings)
Hospitalised, dead (N=45; 93 serial samplings)
Absolute B Lymphocyte counts (cells/mm3)
3000
2000
1000
800
600
400
200
0
**
**
*
**
0 1 2 3 4 5 6 7 8 9 10 11 12 13 14 15 16 17 18 19 20 21 22 23 24 25 26 27 28
Days since admission
No. of analyzed patients
Hospitalised, recovered 22 48 53 65 52 52 40 61 21 30 36 15 19 17 16 6 9 8 7 4 3 7 2 2 6 0 1 2 1
Hospitalised, dead 6 10 10 16 15 6 5 5 3 4 4 1 4 0 2 1 0 1

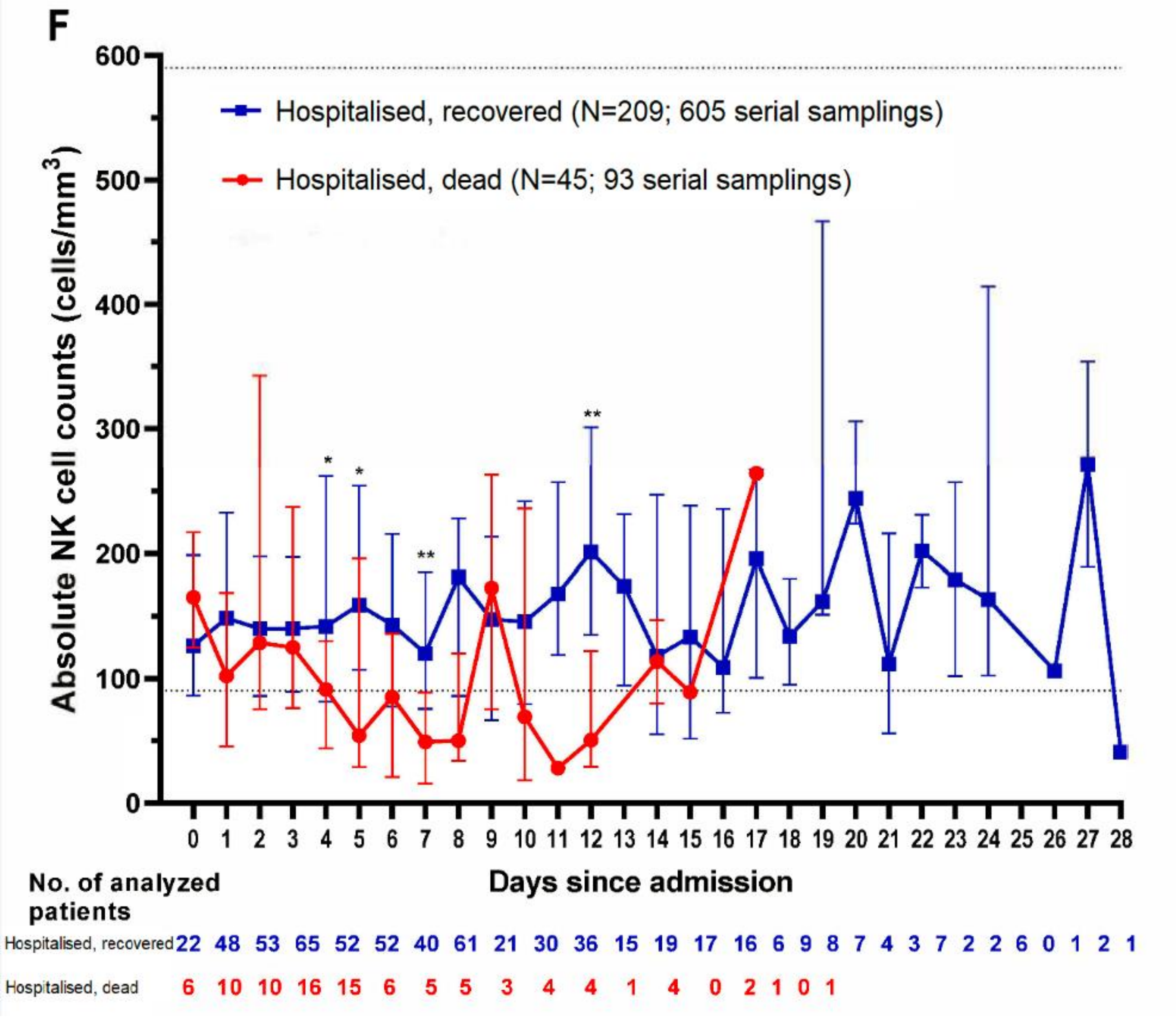
F
Hospitalised, recovered (N=209; 605 serial samplings)
Hospitalised, dead (N=45; 93 serial samplings)
Absolute NK cell counts (cells/mm^3)
600
500
400
300
200
100
0
*
*
**
**
0 1 2 3 4 5 6 7 8 9 10 11 12 13 14 15 16 17 18 19 20 21 22 23 24 25 26 27 28
Days since admission
No. of analyzed patients
Hospitalised, recovered 22 48 53 65 52 52 40 61 21 30 36 15 19 17 16 6 9 8 7 4 3 7 2 2 6 0 1 2 1
Hospitalised, dead 6 10 10 16 15 6 5 5 3 4 4 1 4 0 2 1 0 1

G

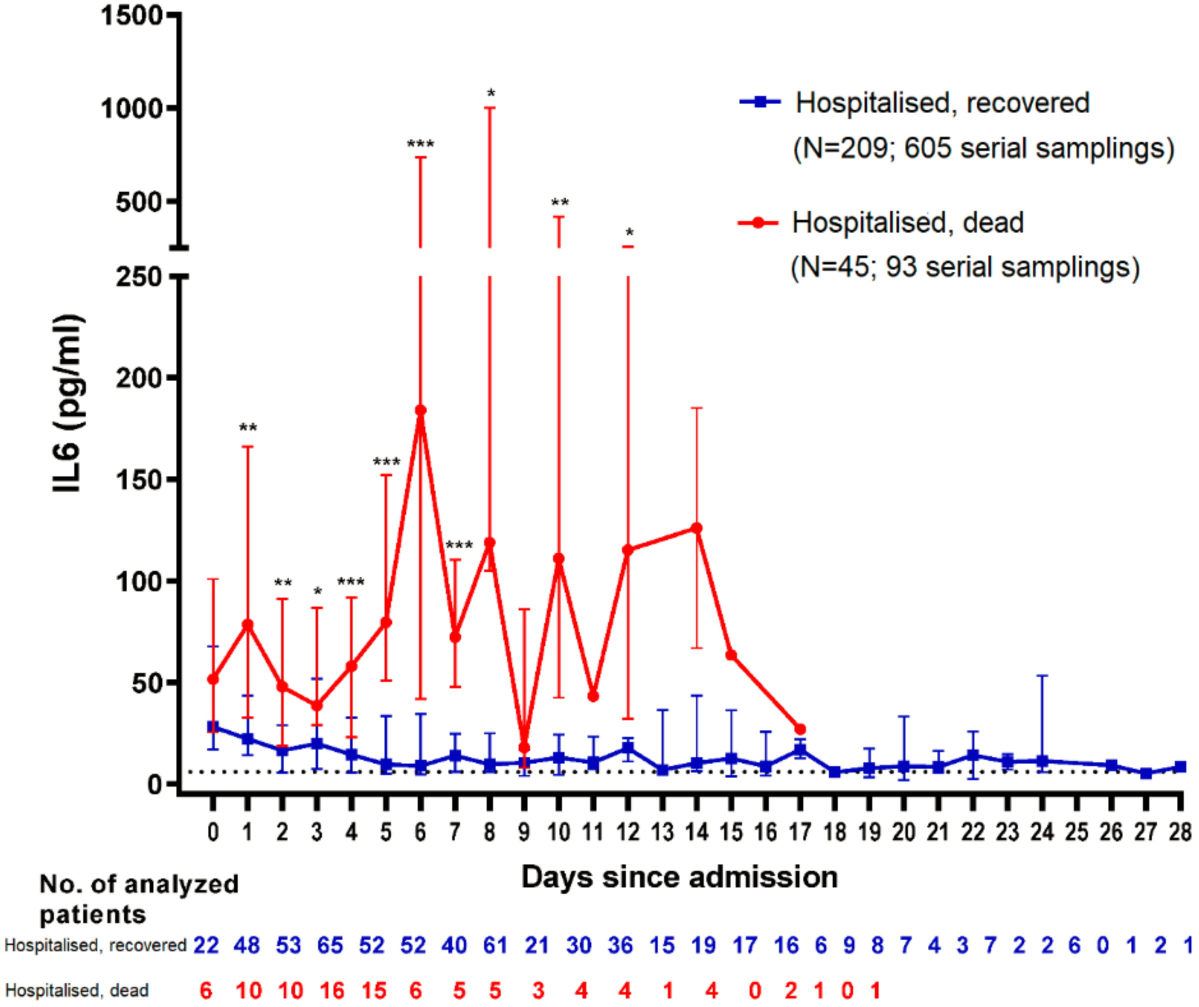
IL6 (pg/ml)
1500
1000
500
250
200
150
100
50
0
0 1 2 3 4 5 6 7 8 9 10 11 12 13 14 15 16 17 18 19 20 21 22 23 24 25 26 27 28
Days since admission
Hospitalised, recovered
(N=209; 605 serial samplings)
Hospitalised, dead
(N=45; 93 serial samplings)
No. of analyzed patients
Hospitalised, recovered 22 48 53 65 52 52 40 61 21 30 36 15 19 17 16 6 9 8 7 4 3 7 2 2 6 0 1 2 1
Hospitalised, dead 6 10 10 16 15 6 5 5 3 4 4 1 4 0 2 1 0 1

Figure E3. The lowest T cells, CD4 and CD8 subpopulations of T cells, B and NK cells absolute counts, and the highest serum IL-6 after admission to the hospital according to whether COVID-19 patients progressed to the primary endpoint of death at 28 days or recovered to hospital discharge. Non-hospitalized controls represent adult individuals with prior COVID-19 confirmed by a nasopharyngeal swab PCR test, post-sampling median 60 days after infection.

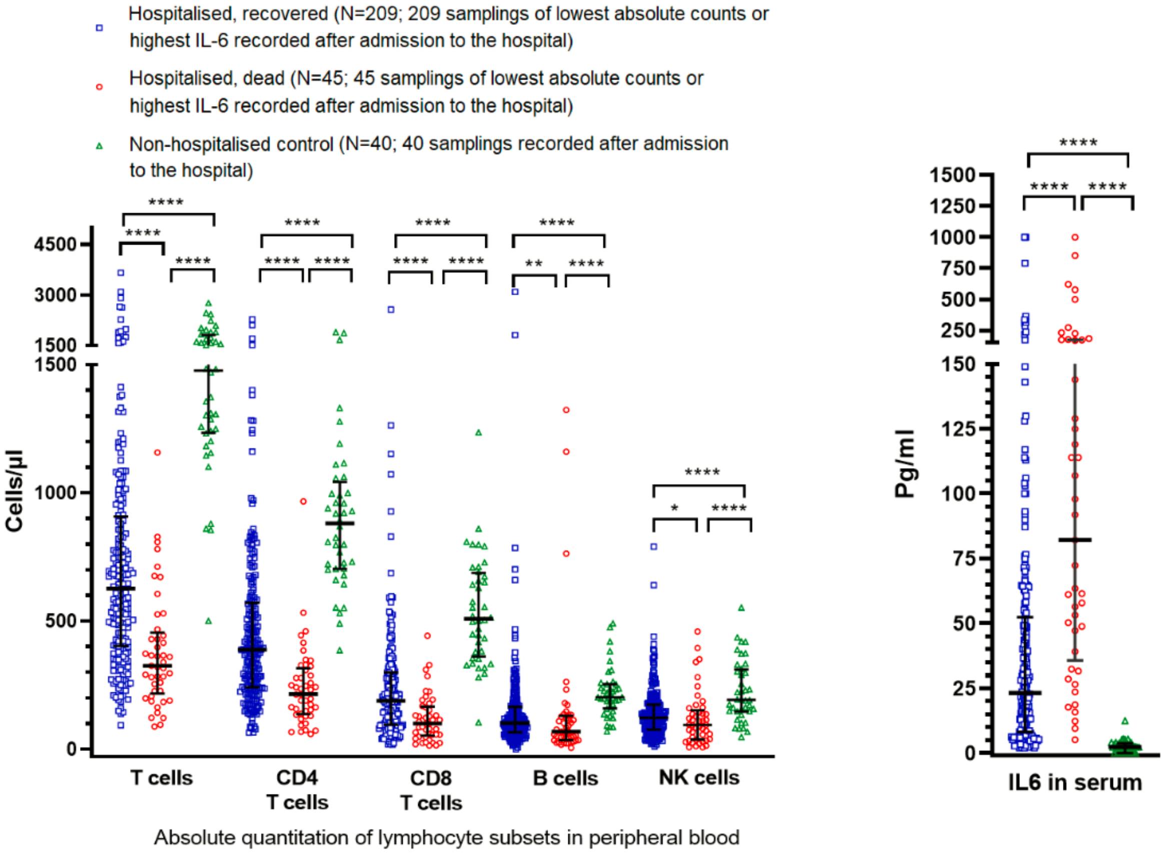

Table E1. Demographic charcteristics, antibody response, lymphocytes, and IL-6 in non-hopsptalized control subjects.

**Age – mean ± SD**	**45.6 ± 12.1**
**Male sex — no. (%)**	22 (55%)
**Anti-SARS-CoV-2 S1 IgG antibodies (pg/ml)**	
**Minimum**	277.2
**25%**	2077
**Median**	2935
**75%**	3046
**Maximum**	3286
**T cells/mm3**	
**Minimum**	501
**25%**	1234
**Median**	1477
**75%**	1814
**Maximum**	2763
**B cells/mm3**	
**Minimum**	69
**25%**	159
**Median**	202
**75%**	253
**Maximum**	491
**NK cells/mm3**	
**Minimum**	47
**25%**	146.5
**Median**	192.5
**75%**	310.5
**Maximum**	552
**IL-6 (pg/ml)**	
**Minimum**	0
**25%**	0
**Median**	2.425
**75%**	3.853
**Maximum**	12.50

Table E2. Associations between demographic and clinical factors, antibody response, lymphocyte subsets, IL-6, and the endpoint of death at 28 days in 254 hospitalized subjects.

**UNIVARIATE COX ANALYSIS**				
	**Hazard ratio**	**Lower 95% CI**	**Upper 95% CI**	**P-value**
**Age — yr**	1.0887	1.0538	1.1247	<0.0001
**Sex (female)**	0.7072	0.3871	1.2920	0.26
**Respiratory support received at the time of the admission (WHO severity ordinal scale 3)[16]**	5.7008	2.5448	12.771	<0.0001
**Type 2 diabetes**	1.2478	0.6713	2.3195	0.484
**Hypertension**	1.0620	0.5813	1.9402	0.845
**No. of coexisting diseases (sum)**	1.2775	0.8435	1.9348	0.247
**Body mass index (≥30.0)§**	0.9399	0.8701	1.0154	0.116
**Glucocorticoid use (Yes)**	0.6615	0.3682	1.1884	0.167
**Anti-SARS-CoV-2 S1 IgG antibodies***	0.9993	0.9990	0.9996	<0.0001
**T cells/mm^3†**	0.9965	0.9951	0.9979	<0.0001
**CD4+ T cells/mm^3†**	0.9948	0.9926	0.9971	<0.0001
**<500 CD4+ cells/mm^3**	8.9102	2.1583	36.7840	0.0025
**<200 CD4+cells/mm^3**	3.8183	2.1246	6.8623	<0.0001
**CD8+ T cells/mm^3†**	0.9936	0.9903	0.9969	0.0002
**B Lymphocyte cells/mm^3†**	0.9998	0.9986	1.0011	0.808
**Natural killer cells/mm^3†**	0.9977	0.9941	1.0014	0.219
**IL-6‡**	1.0022	1.0012	1.0032	<0.0001
**MULTIVARIABLE COX ANALYSIS**				
**Age— yr**	1.0652	1.0294	1.1023	0.0003
**Respiratory support received at the time of admission**	4.5264	1.9594	10.4563	0.0004
**Anti-SARS-CoV-2 spike 1 IgG antibodies***	0.9994	0.9991	0.9996	<0.0001
**T cells/mm^3† or**	0.9977	0.9963	0.9991	0.0015
**CD4+ T cells/mm^3† or**	0.9965	0.9943	0.9988	0.0024
**CD8+ T cells/mm^3†**	0.9960	0.9929	0.9991	0.012
**IL-6‡**	1.0014	1.0001	1.0027	0.031

*SARS-CoV-2 anti S1 IgG antibodies were measured median 4 days after admission to the hospital. †Decreases in circulating lymphocytes were defined as the lowest T cell, CD4, and CD8 subpopulations of T cells, B and NK cell absolute counts recorded after admission. ‡IL-6 increase was defined as the maximal serum level recorded after admission. §BMI data were missing for 34 patients. The decision to prescribe glucocorticoids was at the discretion of the treatment team for each patient. In the multivariable Cox model, only significant predictors (P<0.05) from univariate Cox analysis were retained and only CD3 T-cell counts were used (since they reflect the presence of CD4 as well as CD8 cells). HR-hazard ratio.

Acknowledgments

We thank Monika Šohar, Ines Hasanović and Žan Kogovšek for flow cytometry laboratory support. We thank Eva Bitežnik for data analysing support. We thank Dr Viktorija Tomič, Dr Dane Lužnik, Maruša Ravnik, Vesna Špendal, Mitja Rot, Jerneja Oman and Nataša Debeljak for SARS-CoV-2 PCR testing laboratory support.

References

1. Custovic A, Belgrave D, Lin L, et al. Cytokine Responses to Rhinovirus and Development of Asthma, Allergic Sensitization, and Respiratory Infections during Childhood. *Am J Respir Crit Care Med*. 2018;197(10):1265-1274. doi:10.1164/RCCM.201708-1762OC

2. Garcia-Beltran WF, Lam EC, Astudillo MG, et al. COVID-19-neutralizing antibodies predict disease severity and survival. *Cell*. 2021;184(2):476-488.e11. doi:10.1016/j.cell.2020.12.015

3. Kawasuji H, Morinaga Y, Tani H, et al. Delayed neutralizing antibody response in the acute phase correlates with severe progression of COVID-19. *Sci Rep*. 2021;11(1). doi:10.1038/s41598-021-96143-8

4. Vabret N, Britton GJ, Gruber C, et al. Immunology of COVID-19: Current State of the Science. *Immunity*. Published online 2020. doi:10.1016/j.immuni.2020.05.002

5. Lucas C, Wong P, Klein J, et al. Longitudinal analyses reveal immunological misfiring in severe COVID-19. *Nature*. 2020;584(7821):463-469. doi:10.1038/s41586-020-2588-y

6. Ronit A, Berg RMG, Bay JT, et al. Compartmental immunophenotyping in COVID-19 ARDS: A case series. *J Allergy Clin Immunol*. Published online 2020. doi:10.1016/j.jaci.2020.09.009

7. Huang W, Berube J, McNamara M, et al. Lymphocyte Subset Counts in COVID-19 Patients: A Meta-Analysis. *Cytom Part A*. 2020;97(8):772-776. doi:10.1002/cyto.a.24172

8. Wang F, Nie J, Wang H, et al. Characteristics of peripheral lymphocyte subset alteration in covid-19 pneumonia. *J Infect Dis*. 2020;221(11):1762-1769. doi:10.1093/INFDIS/JIAA150

9. Jiang M, Guo Y, Luo Q, et al. T-Cell Subset Counts in Peripheral Blood Can Be Used as Discriminatory Biomarkers for Diagnosis and Severity Prediction of Coronavirus Disease 2019. *J Infect Dis*. 2020;222(2):198-202. doi:10.1093/infdis/jiaa252

10. Del Valle DM, Kim-Schulze S, Huang HH, et al. An inflammatory cytokine signature predicts COVID-19 severity and survival. *Nat Med*. 2020;26(10):1636-1643. doi:10.1038/s41591-020-1051-9

11. Moore JB, June CH. Cytokine release syndrome in severe COVID-19. *Science (80-)*. 2020;368(6490):473-474. doi:10.1126/science.abb8925

12. McElvaney OJ, McEvoy NL, McElvaney OF, et al. Characterization of the inflammatory response to severe COVID-19 Illness. *Am J Respir Crit Care Med*. 2020;202(6):812-821. doi:10.1164/rccm.202005-1583OC

13. Zhang XX, Tan Y, Ling Y, et al. Viral and host factors related to the clinical outcome of COVID-19. *Nature*. 2020;583(7816):437-440. doi:10.1038/s41586-020-2355-0

14. Horby P, Lim WS, Emberson JR, et al. Dexamethasone in Hospitalized Patients with Covid-19. *N Engl J Med*. 2021;384(8):693-704. doi:10.1056/NEJMoa2021436

15. Beigel JH, Tomashek KM, Dodd LE, et al. Remdesivir for the Treatment of Covid-19 — Final Report. *N Engl J Med*. 2020;383(19):1813-1826. doi:10.1056/nejmoa2007764

16. Tocilizumab in Patients Hospitalized with Covid-19 Pneumonia. *N Engl J Med*. 2021;384(15):1473-1474. doi:10.1056/nejmc2100217
